# On the Functions of the Liver

**Published:** 1851-11

**Authors:** 


*On the Functions of the Liver.* By M. Bernard. — M. Bernard, in a recent course of lectures, delivered at the "College of France," reiterated his views respecting the functions of the liver, and endeavored to confirm them by additional experiments. Although we have already adverted to the various papers he has from time to time published on the subject, a brief recapitulation of the points he believes proved may not be uninteresting, and will be best managed by stating them in the form of propositions.

*On the Formation of Sugar by the Liver.*

1. In an animal that has been prevented access to saccharine and amylaceous food, the blood entering the liver contains no sugar, but that which leaves it always contains it. Dogs fed for six weeks exclusively on meat, present large quantities.

2. It is not only the blood of the organ, but its tissue also, that contains it in abundance.

3. It is found in the livers of all the domestic animals, in birds, fish, reptiles, and even oysters and snails. Towards the fifth month it is found in the fœtal liver, and continues increasing; and it exists even in the embryoes of oviparous animals.

4. The quantity calculated from actual measurement to be contained in the liver of a healthy adult, who was guillotined while fasting, was 23 grammes 267 milligrammes. In a diabetic subject, the liver contained 57 grammes. In the liver of an ox the total quantity was calculated at 243 gr.

5. As a general rule, there is most sugar in the liver of those animals which consume aliments containing sugar. The longer the abstinence the less the quantity.

6. There is more found in adult animals than in young.

7. Although repeated experiments constantly confirm M. Bernard's original assertions that irritation of the olivary bodies of the medulla oblongata induces an almost immediate increase in the quantity of sugar, yet the supposition it did so by irritating the origins of the eighth pair of nerves, is erroneous; for if these nerves be divided, the incretion goes on.

8. Whether the left or right olivary body, or the interval between them, be pierced, the quantity of sugar is alike increased in the urine; but in the first two cases the animal turns continually to the left or the right, and in the last progression occurs in a straight line. The quantity produced, within certain limits, is increased with the size of the instrument used. In rabbits the sugar secretion continues for forty-eight hours after, and in dogs for four or even seven days. The same results follow, whether the animal is fasting or not.

9. During this increased secretion, the animal is in constant agitation; its respiration is accelerated, but its temperature is diminished some degrees. The quantity of urine is increased, besides becoming saccharine.

10. The sugar once produced, whether by the liver or by means of aliment, undergoes destruction in the lungs; but its disappearance is not, like its production, under nervous influence, but is a chemical phenomenon which may take place in contact with air externally to the lungs. The destruction in the lungs give rise to the production of carbonic acid, which is liberated from the air passages. In animals whose olivary processes are pierced, this is given out in larger quantities, and their blood becomes blacker.

11. This diabetic sugar is especially distinguished by the large quantities which can be destroyed by the lungs, being as fifteen to two and a half, as compared with grape sugar. Cane sugar introduced into the blood does not disappear by the lungs, but escapes by the urine.

12. Various circumstances may impede or prevent the secretion of sugar by the liver, as severe pain or lesion of the nervous system (except of the olivary bodies.) Diseases may produce the same effect. Thus sugar has been found to cease being secreted in diabetes during the paroxysm of ague, in pneumonia, &c.

Additional experiments have shown M. Bernard the error of the hypothesis he advanced, that glycosuria was due to an affection of the pneumogastric nerves; and he is now disposed to regard it as due to a special, although unknown alteration of the liver itself, which is indeed generally hypertrophied in this disease.

### *Formation of Fatty Matters by the Liver.*

1. In spite of the great variety of fatty substances, animal and vegetable, the animal that consumes them always produces the same description of fat, owing to the elaboration they have undergone in the economy.

2. To become absorbed, fatty matter must previously have become emulsioned, and the *pancreatic juice* is the fluid by which this is accomplished. Its power of effecting this depends upon an organic matter analogous to fermenta.

3. The amount of fatty matter, introduced as food, is far from explaining the quantity possessed or produced by the individual. There is no fat in the blood which enters the liver, but there is abundance in the blood that leaves it, and therefore its formation within this organ is certain.

4. It is, as in the case of sugar, during digestion that fat is produced in the liver, and after abstinence it disappears. Sometimes it is very abundant, especially in suckling women; whence probably arise the fatty matters of the milk, for the fat of the liver offers most analogy to butter. In such women, the blood itself contains much fat.

5. The fat received in the food after decomposition by the pancreatic juice, and the fat from the liver both enter the blood, and are not entirely destroyed in the lungs, in as much as the arterial blood still contains abundance. As the venous blood contains hardly any, and the vena cava none, we must conclude that the greater part is destroyed in the general capillary system.

6. The production of fat in the liver seems, like that of the production of sugar, to be under the influence of the nervous system; and if this undergoes violent lesion or perturbation, its production ceases. It is remarkable, too, that in proportion as the quantity of sugar increases after puncture of the medulla oblongata, that of the fat diminishes. The same is observed in the diabetes; for from the livers which are loaded with sugar, not an atom of fat can be extracted.

7. Althougn healthy urine has been shown to contain some fat, yet this sometimes predominates so as to constitute a disease under the name of fatty or chylous urine, frequently co-existing with a similar state of the blood. This is probably due to the superabundance of fat secreted by the liver, and constitutes a (so to say) fatty diabetes, just as the excess of sugar does a saccharine one.

8. The products of digestion may thus be said to induce three principal diseases — the sugar, diabetes; the fat, the so-called chylous urine; and albumen, the albuminous diseases.

*Fibrin formed in the Liver.*

1. The blood entering the liver contains little fibrin, and coagulates with difficulty even when the animal is fed on flesh. The fibrin of the aliments is dissolved by the gastric juice, and converted into a matter analogous to albumen, termed by Mialhe, albuminose. But the blood which quits the liver contains much fibrin and therefore the albuminose of the blood of the abdominal veins has become transformed into fibrinous matter.

2. It is during digestion that the blood, traversing the liver, becomes loaded with this abundance of fibrin.

*Secretion of Bile.*

1. The secretion of bile differs from those already named, as it is continuous, while they occur only during digestion.

2. The bile is not a mere excrementitious fluid, but influences digestion usefully, contributing with the gastric and pancreatic juices to constitute that most powerful solvent — the intestinal liquid.

3. The bile seems to be essentially endowed with anti-putrefactive properties. It regulates the chemical reactions occurring during digestion, prevents fermentation, and opposes the formation of the gases which result from the decomposition of azotised and non-azotised aliments.

4. When bile is prevented reaching the intestine, putrefactive fermentation, no longer opposed by the acids of this fluid, induces diarrhœa; which may also be induced by a predominance of alkali in the intestines, and may be under such circumstances advantageously treated by acids.

*Resume.* — From what has preceded, it is evident that sugar, fat and fibrin, are fabricated in the liver, and that whatever the alimentation may be, this organ transforms it into appropriate matter of nutrition, so that the great variety of food taken does not derange the composition of the blood and prevent its being identical. In a chemical point of view, the organ is then of great consequence, and is properly regarded as one of sanguification. While in the herbivora, whose aliments furnish much sugar, this secretory organ furnishes least sugar, in the carnivora, who ingest much fatty matter, it secretes least fat. So, too, the liver forms fibrin abundantly, in proportion as the vena portæ contains less. The liver, therefore, while it is an organ of sanguification, is always one for adjusting the equilibrium.

It the above products furnished by the liver serve to keep the circulating fluid in a composition essentially fit for nutrition, the bile, acting in opposite direction, contributes to the same end, by removing the principles that are in excess, especially carbon; so that the liver must be considered not only as an organ of sanguification and equilibrium, but also one exerting a depurative action on the blood.

### *Circulation of the Liver.*

1. Two powerful causes — the pressure of the abdominal viscera and the venous aspiration — contribute to this; but other contrivances are required to meet the varying degrees of plenitude to which the organ is liable.

2. Certain vessels, seen easiest in the horse, communicate directly between the vena portæ and vena cava, conveying a portion of the blood without its having undergone modification in the capillary circulation of the liver, and operating as a kind of diverticulum, preventing the organ from being too greatly and too suddenly engorged.

3. Congestion of the liver and heart, in the case of unusual afflux of fluids, is further provided against by the agency of a special hepatic-renal circulation, not so distinct in man, in whom the liver is liable to become congested by over-exertion, as in the horse, in whose liver this does not produce the same effect.

4. The active and abundant circulation through the liver is an important source of animal heat, the blood leaving the organ having acquired an additional temperature of about two degrees Fahrenheit. — *L'Union Medicale,* 1850, Nos. 82, 85, 88, 91, 98, 103, 106, 113, 115.

